# Functional Oral Intake, Eating-Related Quality of Life and Oral Health-Related Quality of Life in Older Adults with Stroke: A Cross-Sectional Study

**DOI:** 10.3390/healthcare14182970

**Published:** 2026-09-11

**Authors:** Burak Manay, Mustafa İbas, Özcan Sönmez, Mehmet Nuri Elgörmüş, Mehmet Şerif Önen, Gizem İbas, Hakan Parlak, Murat Ünsel, Alperen Şentürk, Ramazan Güven, Ramazan Ocal

**Affiliations:** 1 Department of Speech and Language Therapy, Faculty of Health Sciences, Istanbul Atlas University, 34403 Istanbul, Turkey; mehmetnuri.elgormus@atlas.edu.tr (M.N.E.); alperen.senturk@atlas.edu.tr (A.Ş.); 2 Department of Otolaryngology, Head and Neck Surgery, Istanbul Atlas University, 34403 Istanbul, Turkey; mustafa.ibas@atlas.edu.tr; 3 Department of Internal Medicine, Faculty of Medicine, Istanbul Atlas University, 34403 Istanbul, Turkey; ozcan.sonmez@atlas.edu.tr; 4 Department of Physical Medicine and Rehabilitation, Istanbul Atlas University, 34403 Istanbul, Turkey; serif.onen@atlas.edu.tr; 5 Department of Otolaryngology, Bağcılar Training and Research Hospital, University of Health Sciences, 34200 Istanbul, Turkey; ibasgizem@gmail.com; 6 Department of Anesthesiology and Reanimation, Faculty of Medicine, İstinye University, 34010 Istanbul, Turkey; hakan.parlak@istinye.edu.tr; 7 Department of Anesthesiology and Reanimation, Division of Intensive Care Medicine, Faculty of Medicine, Istanbul Atlas University, 34403 Istanbul, Turkey; muratunsel@hotmail.com; 8 Department of Emergency Medicine, Faculty of Medicine, Istanbul Atlas University, 34403 Istanbul, Turkey; ramazan.guven@atlas.edu.tr; 9 Department of Otolaryngology, Head and Neck Surgery, Ankara Training and Research Hospital, University of Health Sciences, 06230 Ankara, Turkey; drramazanocal@gmail.com

**Keywords:** stroke, dysphagia, oral health, oral health-related quality of life

## Abstract

**Highlights:**

**What are the main findings?**

- Higher functional oral intake was associated with better oral health-related quality of life.
- A statistically supported total indirect association between functional oral intake and GOHAI was observed through Desire to Eat, Food Selection, and Eating Time considered collectively.

**What are the implications of the main findings?**

- Eating-related experiences may warrant consideration in patient-centred post-stroke dysphagia care.
- Oral health-related quality of life should be considered alongside swallowing function and functional oral intake in older adults with stroke.

**Abstract:**

**Background/Objectives:** Oral health is important for safe oral feeding after stroke. However, the relationships among oral health-related quality of life, functional oral intake, and eating-related quality of life remain unclear. This study examined these associations in older adults with stroke. **Methods:** This cross-sectional observational study included 100 adults aged ≥65 years with stroke receiving oral feeding (FOIS ≥ 4). Oral health-related quality of life was assessed using the Geriatric Oral Health Assessment Index (GOHAI) and Oral Health Impact Profile-14 (OHIP-14), and eating-related quality of life using selected Swallowing Quality of Life Questionnaire (SWAL-QOL) domains. Correlation, multiple linear regression, and exploratory parallel indirect-effect analyses were performed. **Results:** GOHAI was most strongly correlated with Desire to Eat (r = 0.632, *p* < 0.001), whereas OHIP-14 showed the strongest negative correlation with Desire to Eat (r = −0.603, *p* < 0.001). FOIS was positively associated with GOHAI (ρ = 0.487, *p* < 0.001) and negatively associated with OHIP-14 (ρ = −0.571, *p* < 0.001). Desire to Eat, Food Selection, and Eating Time were independently associated with GOHAI. The exploratory parallel analysis showed a statistically supported total indirect association between FOIS and GOHAI through these eating-related domains collectively (indirect effect = 2.7496, 95% bootstrap CI 0.6070 to 4.9102), while no specific indirect effect was individually supported. **Conclusions:** Functional oral intake, eating-related experiences, and oral health-related quality of life were significantly interrelated. The exploratory findings suggest a collective statistical indirect association through eating-related domains without establishing temporal or causal mediation.

## 1. Introduction

Stroke is a leading cause of mortality and long-term disability worldwide, and its burden is expected to increase with population ageing [1,2]. Dysphagia is a common complication after stroke and is associated with aspiration pneumonia, malnutrition, dehydration, prolonged hospitalization and mortality [3]. Even among individuals receiving oral feeding, swallowing difficulties may restrict food selection, prolong mealtimes and reduce eating-related quality of life [4].

Safe oral feeding depends on swallowing function and good oral health. Oral health supports essential orofacial functions, including chewing, swallowing and speaking, and plays an important role in safe oral feeding [5]. Age-related oral conditions, stroke-related orofacial motor impairments, and reduced self-care abilities may coexist with poorer oral health and swallowing difficulties after stroke [6,7,8]. Importantly, the relationship between oral health, orofacial function, and dysphagia should be considered multidirectional rather than a simple causal relationship. Oral health interventions may improve swallowing outcomes and reduce aspiration pneumonia, highlighting oral health as an important component of dysphagia rehabilitation [9,10]. Oral health also influences quality of life beyond swallowing outcomes.

Oral health-related quality of life reflects the impact of oral conditions on functional ability, psychosocial well-being and social participation and is an important patient-reported outcome [11]. Systematic reviews have shown that it is impaired after stroke and associated with swallowing difficulties and functional dependency [9]. Likewise, dietary modifications, prolonged mealtimes and avoidance of preferred foods negatively affect eating experiences and quality of life, although these patient-centred outcomes remain less studied than physiological swallowing measures [4,12].

Oral and orofacial pain represents another clinically relevant dimension of oral health-related quality of life, as pain and discomfort may adversely affect eating experiences and perceived oral function. A recent meta-analysis reported a global prevalence of 19% for concurrent oral and facial pain, 21% for oral pain, and 12% for facial pain alone; notably, the prevalence of concurrent oral and facial pain was approximately 10% among adults aged >60 years [13]. These findings further highlight the relevance of pain and discomfort when considering patient-reported oral health-related quality of life in older populations.

Although oral health-related quality of life, functional oral intake, and eating-related quality of life have each been investigated after stroke, few studies have examined these factors simultaneously in older adults. Functional oral intake reflects the extent to which an individual is able to consume food and liquids orally, whereas Desire to Eat, Food Selection, and Eating Time capture distinct patient-reported aspects of the eating experience, including motivation to eat, perceived limitations in food choice, and the time required for meals. These domains may therefore provide complementary information about how differences in functional oral intake are statistically associated with perceived oral health-related quality of life. However, because these relationships may be bidirectional and cannot be temporally ordered in a cross-sectional design, they should not be interpreted as causal mechanisms. To address these gaps, the present study investigated the associations among functional oral intake, oral health-related quality of life, and eating-related quality of life in older adults (≥65 years) with stroke receiving oral feeding. Exploratory indirect-effect analyses were additionally conducted to examine statistical indirect associations through Desire to Eat, Food Selection, and Eating Time, without assuming temporal or causal relationships.

Accordingly, the primary objective of this study was to examine the associations between functional oral intake, eating-related quality-of-life domains, and oral health-related quality of life in older adults with stroke. We hypothesized that higher functional oral intake would be associated with better oral health-related quality of life and more favorable scores across the selected eating-related domains of the Swallowing Quality of Life Questionnaire (SWAL-QOL). As secondary objectives, we examined which eating-related domains were independently associated with oral health-related quality of life after accounting for age, neurological severity, and functional oral intake. We hypothesized that eating-related domains would show independent associations with oral health-related quality of life beyond these clinical and demographic factors. Finally, as an exploratory analysis, we examined whether the association between functional oral intake and the Geriatric Oral Health Assessment Index (GOHAI) was characterized by statistical indirect associations through Desire to Eat, Food Selection, and Eating Time. Given the cross-sectional design, this indirect-effect analysis was considered exploratory and was not intended to establish temporal or causal mediation.

## 2. Methods

### 2.1. Study Design and Setting

This single-centre cross-sectional observational study was conducted at Istanbul Atlas University Hospital to investigate the relationships among functional oral intake, eating-related quality of life, and oral health-related quality of life in older adults with stroke receiving oral feeding (Functional Oral Intake Scale [FOIS] ≥ 4). This study was reported in accordance with the STROBE guidelines (Table S1). Consecutive participants were recruited from the Neurology and Physical Medicine and Rehabilitation outpatient clinics between April and July 2026 after meeting the eligibility criteria. All demographic and clinical data were collected during a single study visit using standardized assessment tools. No invasive procedures or therapeutic interventions beyond routine clinical practice were performed. Study assessments were questionnaire- and scale-based and did not involve the administration of food or liquid boluses or swallowing provocation procedures. The study was approved by the Istanbul Atlas University Non-Interventional Scientific Research Ethics Committee (Meeting No. 03, Decision No. 76; Approval Date: 23 March 2026; Ref. No. E-22686390-050.99-96360) and conducted in accordance with the Declaration of Helsinki. Capacity to provide informed consent was assessed based on the participant’s ability to understand the study information, communicate a voluntary decision, and meet the predefined cognitive eligibility criterion (MMSE ≥ 24). Written informed consent was obtained from all participants before enrolment. Participant data were coded using unique study identifiers, and directly identifying information was not included in the analytical dataset. Study data were stored securely and were accessible only to the research team.

### 2.2. Study Population

A total of 100 adults aged ≥65 years with clinically and radiologically confirmed first-ever ischemic or haemorrhagic stroke who were receiving oral feeding (FOIS ≥ 4) were consecutively recruited from the Neurology and Physical Medicine and Rehabilitation outpatient clinics of Istanbul Atlas University Hospital between April and July 2026.

Inclusion criteria were: (1) age ≥ 65 years; (2) first-ever ischemic or haemorrhagic stroke; (3) self-reported dysphagia (Eating Assessment Tool-10 [EAT-10] score ≥ 3); (4) oral feeding (FOIS ≥ 4); (5) Mini-Mental State Examination (MMSE) score ≥ 24; and (6) written informed consent. The MMSE ≥ 24 criterion was used to ensure sufficient cognitive capacity for reliable comprehension and completion of the patient-reported questionnaires used in the study.

Exclusion criteria were: (1) history of head and neck cancer; (2) neurodegenerative disorders (Parkinson’s disease, amyotrophic lateral sclerosis, or dementia); (3) severe aphasia or communication disorders preventing questionnaire completion; (4) active psychiatric disorders or acute systemic infection; and (5) refusal or inability to provide informed consent.

### 2.3. Data Sources

Patients with a confirmed diagnosis of ischemic or haemorrhagic stroke (International Classification of Diseases, Tenth Revision [ICD-10] codes I63 and I61) were recruited from the Neurology and Physical Medicine and Rehabilitation outpatient clinics of Istanbul Atlas University Hospital. Demographic and clinical data were collected using a standardized participant information form during the study visit. Swallowing function, cognitive status, swallowing-related quality of life, and oral health-related quality of life were assessed using standardized clinical instruments. The GOHAI, OHIP-14 and SWAL-QOL were administered face-to-face using paper-based forms. All questionnaire responses were provided directly by the participants themselves, without caregiver assistance or proxy responses. Participants were asked to complete all questionnaire items. Immediately after completion, the forms were reviewed by the researcher for completeness and data quality and any inadvertently omitted items were identified and completed by the participant before the end of the study visit.

### 2.4. Assessment Procedures

All assessments were performed during a single outpatient visit by two experienced speech and language therapists. After written informed consent, demographic and clinical information were collected using a structured participant information form. Cognitive function was screened using the MMSE, and self-reported swallowing symptoms were assessed using the EAT-10, with a score ≥ 3 used as an eligibility criterion. Functional oral intake, swallowing-related quality of life, and oral health-related quality of life were assessed using the FOIS, SWAL-QOL, and GOHAI and Oral Health Impact Profile-14 (OHIP-14), respectively. GOHAI and OHIP-14 were administered because they assess complementary dimensions of oral health-related quality of life. GOHAI primarily evaluates functional limitations, pain, and discomfort associated with oral conditions in older adults, whereas OHIP-14 provides a broader assessment of the psychosocial impact of oral conditions. Stroke severity was assessed using the National Institutes of Health Stroke Scale (NIHSS).

#### 2.4.1. Demographic and Clinical Characteristics

Demographic and clinical characteristics were collected using a standardized data collection form. Recorded variables included age, sex, education level, marital status, stroke type, time since stroke, denture use, oral hygiene habits, oral pain or discomfort, difficulty during eating, coughing or choking while eating, and avoidance of specific foods due to swallowing or self-reported oral problems. Stroke severity was assessed using the NIHSS. Cognitive function was evaluated using the MMSE, and only participants with MMSE scores ≥ 24 were included.

#### 2.4.2. Functional Oral Intake Scale (FOIS)

FOIS was developed by Crary et al. to classify the functional level of oral intake in individuals with dysphagia [14]. The scale consists of seven levels ranging from Level 1 (nothing by mouth) to Level 7 (total oral diet with no restrictions), with higher scores indicating better functional oral intake. In the present study, FOIS was used to determine participants’ oral intake level and as an eligibility criterion, with only patients scoring FOIS ≥ 4 being included in the study.

#### 2.4.3. Swallowing Quality of Life Questionnaire (SWAL-QOL)

SWAL-QOL was developed to assess swallowing-related quality of life in adults with oropharyngeal dysphagia [12]. The questionnaire consists of 44 items, with domain scores ranging from 0 to 100 and higher scores indicating better swallowing-related quality of life. The Turkish version has demonstrated good validity and reliability in individuals with dysphagia [15].

Consistent with the study objectives, analyses focused on the Desire to Eat, Food Selection, Eating Time, Fear and Social domains because of their relevance to eating experiences and eating-related quality of life. The selected domains were analysed individually as specific aspects of eating-related quality of life; they were not combined or interpreted as a separately validated composite measure, and no overall SWAL-QOL score was calculated.

#### 2.4.4. Geriatric Oral Health Assessment Index (GOHAI)

GOHAI was developed by Atchison and Dolan to assess oral health-related quality of life in older adults [16]. The questionnaire consists of 12 items rated on a Likert-type scale. Total scores range from 0 to 60, with higher scores indicating better perceived oral health and oral health-related quality of life [17]. The Turkish version has demonstrated good validity and reliability in adults aged ≥65 years [18]. In this study, the total GOHAI score was used to evaluate oral health-related quality of life.

#### 2.4.5. The Oral Health Impact Profile-14 (OHIP-14)

OHIP-14 was used to assess oral health-related quality of life. It is the 14-item short version of the original Oral Health Impact Profile [19]. The Turkish version has demonstrated good validity, reliability and comprehensibility [20]. Items are scored on a 5-point Likert scale (0 = never to 4 = very often), yielding total scores ranging from 0 to 56, with higher scores indicating poorer oral health-related quality of life.

### 2.5. Statistical Analysis

An a priori sample-size calculation was performed using G*Power software (version 3.1.9.7) before study initiation. For the multiple linear regression analysis with eight predictors, assuming a medium effect size (f^2^ = 0.15), an alpha level of 0.05, and 80% statistical power, the required sample size was estimated as 109 participants. Due to the number of eligible participants available during the predefined recruitment period, the final sample consisted of 100 participants.

Statistical analyses were performed using IBM SPSS Statistics version 30.0 (IBM Corp., Armonk, NY, USA). Continuous variables are presented as mean ± standard deviation (SD), and categorical variables as frequencies and percentages. Normality was assessed using the Shapiro–Wilk test and skewness and kurtosis values. Although the Shapiro–Wilk test indicated deviations from normality for several variables, skewness and kurtosis values were within the acceptable range (±1); therefore, parametric analyses were performed where appropriate. There were no missing data for the variables included in the analyses; therefore, no imputation was required.

Correlation analyses were performed to examine bivariate associations among the study variables. Pearson correlation coefficients were used for GOHAI, OHIP-14, and the selected SWAL-QOL domains, whereas associations involving the ordinal FOIS were assessed using Spearman’s rank correlation coefficient (ρ). Correlation coefficients are reported with 95% confidence intervals (CIs). For interpretation of Pearson correlation effect sizes, absolute r values of 0.20, 0.40, and 0.70 were considered indicative of small, medium, and large effects, respectively, in accordance with the recommended guidelines for dental research [21].

Multiple linear regression analyses were performed to identify variables independently associated with GOHAI and OHIP-14. Separate models were constructed for each outcome, with age, NIHSS, FOIS, Desire to Eat, Food Selection, Eating Time, Fear, and Social entered simultaneously using the Enter method. Regression coefficients, standard errors, standardized coefficients (β), t values, *p* values, 95% confidence intervals for the unstandardized regression coefficients (B), tolerance, and variance inflation factor (VIF) values were reported. Regression assumptions were assessed using residual diagnostic plots. Residual normality was evaluated using histograms and normal P–P plots, while linearity and homoscedasticity were assessed by examining standardized residuals against predicted values. No substantial violations of these assumptions were observed. Multicollinearity was assessed using tolerance and VIF values.

An exploratory parallel indirect-effect analysis was performed using PROCESS macro version 4.2 (Model 4) to examine the statistical indirect association between FOIS and GOHAI through Desire to Eat, Food Selection, and Eating Time entered simultaneously as parallel intermediate variables. The total indirect effect and the specific indirect effects for each domain were estimated using 5000 bias-corrected bootstrap samples. An indirect effect was considered statistically supported when its 95% bootstrap confidence interval did not include zero. Given the cross-sectional design, this analysis was considered exploratory and was interpreted as an assessment of statistical indirect associations rather than evidence of temporal or causal mediation.

No formal adjustment for multiple comparisons was applied because the analyses were hypothesis-driven and focused on prespecified study variables; therefore, the findings should be interpreted with consideration of the potential for inflated type I error. A two-sided *p* < 0.05 was considered statistically significant.

## 3. Results

### 3.1. Participant Characteristics

A total of 100 older adults with stroke receiving oral feeding were included in the study. The mean age of the participants was 72.08 ± 7.36 years and 51.0% were male. Ischemic stroke was present in 59.0% of the participants, while 41.0% had haemorrhagic stroke. The mean time since stroke was 5.20 ± 3.77 months, with mean NIHSS and FOIS scores of 17.00 ± 9.36 and 5.94 ± 1.20, respectively. The mean scores for the SWAL-QOL domains, GOHAI and OHIP-14 are presented in Table 1.

### 3.2. Correlations Between Oral Health-Related Quality-of-Life Measures and Eating-Related Quality of Life

Pearson correlation analysis showed that GOHAI total score was significantly and positively correlated with all selected SWAL-QOL domains, with the strongest correlation observed for Desire to Eat (r = 0.632, 95% CI 0.498 to 0.737, *p* < 0.001). Conversely, OHIP-14 total score was significantly and negatively correlated with all SWAL-QOL domains, with the strongest association also observed for Desire to Eat (r = −0.603, 95% CI −0.713 to −0.464, *p* < 0.001). Furthermore, GOHAI and OHIP-14 total scores were strongly and negatively correlated (r = −0.804, 95% CI −0.865 to −0.720, *p* < 0.001). Detailed correlation results are presented in Table 2.

### 3.3. Correlations Between Functional Oral Intake and Oral Health-Related Quality of Life/Eating-Related Quality of Life

Spearman correlation analysis demonstrated that FOIS score was significantly and positively correlated with GOHAI (ρ = 0.487, 95% CI 0.322 to 0.624, *p* < 0.001) and all selected SWAL-QOL domains, with the strongest correlation observed for Desire to Eat (ρ = 0.504, 95% CI 0.344 to 0.635, *p* < 0.001). Conversely, FOIS score was significantly and negatively correlated with OHIP-14 (ρ = −0.571, 95% CI −0.690 to −0.423, *p* < 0.001). Detailed correlation results are presented in Table 3.

### 3.4. Multiple Linear Regression Analysis

Multiple linear regression analyses using the Enter method were performed to identify variables independently associated with oral health-related quality of life. In the GOHAI model, Desire to Eat (β = 0.249, B = 0.144, 95% CI 0.017 to 0.272, *p* = 0.027), Food Selection (β = 0.179, B = 0.123, 95% CI 0.008 to 0.238, *p* = 0.037), and Eating Time (β = 0.285, B = 0.165, 95% CI 0.058 to 0.272, *p* = 0.003) were independently associated with GOHAI, whereas age, NIHSS, FOIS, Fear, and Social were not statistically significant (*p* > 0.05). The overall model was statistically significant (R^2^ = 0.558, adjusted R^2^ = 0.519, F(8, 91) = 14.377, *p* < 0.001).

In the OHIP-14 model, FOIS (β = −0.304, B = −4.324, 95% CI −6.604 to −2.043, *p* < 0.001), Food Selection (β = −0.217, B = −0.145, 95% CI −0.249 to −0.040, *p* = 0.007), and Eating Time (β = −0.300, B = −0.169, 95% CI −0.266 to −0.072, *p* = 0.001) were independently associated with OHIP-14, whereas age, NIHSS, Desire to Eat, Fear, and Social were not statistically significant (*p* > 0.05). The overall model was statistically significant (R^2^ = 0.618, adjusted R^2^ = 0.584, F(8, 91) = 18.403, *p* < 0.001). Detailed regression results are presented in Table 4.

### 3.5. Exploratory Indirect-Effect Analysis

An exploratory indirect-effect analysis using PROCESS Model 4 was conducted with Desire to Eat, Food Selection, and Eating Time entered simultaneously as parallel mediators. FOIS was significantly associated with Desire to Eat (B = 7.6166, *p* = 0.002), Food Selection (B = 6.3432, *p* = 0.003), and Eating Time (B = 6.2194, *p* = 0.011). In the outcome model, Desire to Eat (B = 0.1824, *p* = 0.003) and Food Selection (B = 0.1317, *p* = 0.047) were significantly associated with GOHAI, whereas Eating Time was not (B = 0.0862, *p* = 0.160). The total effect of FOIS on GOHAI was significant (B = 4.8679, *p* = 0.001), whereas the direct effect after inclusion of the parallel mediators was not statistically significant (B = 2.1183, *p* = 0.110). The total indirect effect was statistically supported because the 95% bootstrap confidence interval did not include zero (indirect effect = 2.7496, BootSE = 1.0752, 95% bootstrap CI: 0.6070 to 4.9102). However, the 95% bootstrap confidence intervals for the specific indirect effects through Desire to Eat, Food Selection, and Eating Time each included zero. Therefore, the findings indicate an overall statistical indirect association across the eating-related domains rather than a statistically supported specific indirect effect through any single domain. Detailed results are presented in Table 5.

## 4. Discussion

The present study showed that functional oral intake was associated with oral health-related quality of life and eating-related experiences in older adults with stroke receiving oral feeding. GOHAI and OHIP-14 were significantly associated with the selected SWAL-QOL domains, and functional oral intake was related to both oral health-related quality-of-life measures and the selected eating-related domains. These findings are consistent with previous evidence reporting associations among swallowing-related function, eating-related experiences, and oral health-related quality of life after stroke [9,22]. In the exploratory parallel indirect-effect analysis, the total indirect association between FOIS and GOHAI through Desire to Eat, Food Selection, and Eating Time was statistically supported. These findings indicate interrelationships among functional oral intake, eating-related experiences, and oral health-related quality of life in this population, without establishing their temporal or causal ordering.

The associations between oral health-related quality of life and the selected SWAL-QOL domains indicate that patient-reported oral health impact is related to eating experiences after stroke. Previous studies have reported associations among impaired chewing ability, oral discomfort, reduced numbers of occluding tooth pairs, swallowing difficulties, and poorer oral health-related quality of life in older adults [9,23,24,25]. However, these clinical oral characteristics were not objectively assessed in the present study and therefore cannot be inferred from the GOHAI or OHIP-14 findings. Accordingly, the observed associations should be interpreted as relationships between patient-reported oral health-related quality of life and eating-related experiences rather than as evidence of objective oral health or masticatory status.

Oral and orofacial pain may also be relevant when interpreting patient-reported oral health-related quality of life, given its substantial prevalence in adult and older populations [13]. Pain or discomfort may be associated with eating motivation, food selection, mealtime experiences, and perceived oral function. Because the present analyses did not evaluate oral or orofacial pain as a separate predictor or potential confounder, its specific contribution to the observed associations cannot be determined. Future studies incorporating dedicated measures of oral and orofacial pain alongside objective dental assessments may help clarify these relationships.

The significant associations between functional oral intake and both oral health-related and eating-related quality of life are consistent with previous studies reporting relationships between functional oral intake, swallowing-related quality of life, participation in everyday eating activities, and eating-related experiences [22,26,27,28,29]. Improvements in oral function during stroke rehabilitation have also been reported alongside better functional oral intake [29]. In the present cross-sectional sample, higher FOIS scores were associated with more favorable eating-related experiences and oral health-related quality of life; however, the direction of these relationships cannot be established. These findings therefore support considering functional oral intake and patient-reported eating experiences as related dimensions of post-stroke functioning rather than implying that better oral intake directly improves oral health-related quality of life or psychosocial well-being.

The multivariable analyses showed that several dimensions of eating-related quality of life were independently associated with oral health-related quality of life after accounting for age, stroke severity, and functional oral intake. Specifically, Desire to Eat, Food Selection and Eating Time were independently associated with GOHAI, whereas functional oral intake, Food Selection and Eating Time were independently associated with OHIP-14. NIHSS was not independently associated with either oral health-related quality-of-life outcome in the multivariable models. However, this finding should not be interpreted as evidence that eating-related experiences are more important than neurological severity, as the absence of an independent association may reflect sample characteristics, the distribution of NIHSS scores, statistical power or correlations among the variables included in the models. According to the predefined effect-size thresholds, the observed Pearson correlations were predominantly medium in magnitude, while the correlation between GOHAI and OHIP-14 was large. The independent associations identified in the multivariable models were more modest. Statistical significance should therefore be distinguished from clinical relevance, particularly because the present study did not establish thresholds for clinically meaningful differences in oral health-related quality of life. Accordingly, the clinical importance of these associations requires confirmation in prospective studies. Consistent with previous studies, oral health-related quality of life is influenced not only by oral conditions and swallowing function but also by mealtime experiences [7,9,26,27,28]. Likewise, interventions targeting meal presentation, food texture and visual appeal improve willingness to eat and eating-related quality of life in older adults with dysphagia [4]. The differing patterns of associations observed for GOHAI and OHIP-14 may reflect differences in the dimensions of oral health-related quality of life captured by these instruments; however, these differences should be interpreted cautiously. Some conceptual overlap among the selected SWAL-QOL domains, GOHAI, and OHIP-14 should also be considered when interpreting these associations. Although the instruments assess distinct constructs, they may capture related patient-reported experiences concerning eating, oral function, discomfort, and psychosocial impact. Importantly, the absence of problematic statistical multicollinearity based on VIF values does not eliminate the possibility of conceptual overlap among these measures. Consequently, part of the observed associations, particularly in the multivariable models, may reflect shared patient-reported dimensions rather than entirely independent constructs.

The exploratory parallel indirect-effect analysis provided evidence of a total statistical indirect association between FOIS and GOHAI through Desire to Eat, Food Selection, and Eating Time considered jointly. However, the specific indirect effects should be interpreted according to their individual bootstrap confidence intervals rather than as evidence that any single eating-related domain represents a specific explanatory mechanism. The observed pattern is broadly consistent with previous research showing associations between swallowing difficulties, eating enjoyment, meal satisfaction, and eating-related quality of life [4,27,28]. Previous research has described associations between swallowing impairment and nutritional, behavioural, and patient-reported quality-of-life outcomes [8]. In the present study, the exploratory indirect-effect findings further demonstrated statistical interrelationships among functional oral intake, eating-related experiences and oral health-related quality of life, without identifying a causal or explanatory pathway. Alternative causal structures are equally plausible; for example, poorer oral health-related quality of life may adversely influence eating-related experiences, which may in turn be associated with reduced functional oral intake. Therefore, the ordering examined in the exploratory indirect-effect model should be regarded as one possible statistical representation of these interrelationships rather than evidence supporting a particular directional pathway. From a clinical perspective, eating motivation and mealtime experiences may warrant consideration alongside swallowing safety within patient-centred multidisciplinary dysphagia care [30,31]. Multidisciplinary stroke rehabilitation teams may consider assessing patient-reported difficulties related to eating motivation, food selection, mealtime duration, and oral health-related quality of life, which may help identify individuals requiring broader assessment and coordinated input from speech and language therapists, physicians, dietitians, and dental professionals, as appropriate. However, these findings should not be interpreted as evidence that interventions targeting these factors will improve patient outcomes; prospective and interventional studies are needed to determine their clinical utility and potential effects on nutritional, psychosocial, and oral health-related quality-of-life outcomes.

This study has several strengths. To the best of our knowledge, it is among the first to simultaneously examine the associations among functional oral intake, oral health-related quality of life, and eating-related quality of life in older adults with stroke. The combined use of two validated oral health-related quality-of-life instruments and selected SWAL-QOL domains provided a comprehensive patient-centred assessment. In addition, the exploratory indirect-effect analysis allowed for examination of whether the association between functional oral intake and GOHAI was statistically characterized by indirect associations through Desire to Eat, Food Selection, and Eating Time, without implying a temporal or causal mechanism.

Nevertheless, several limitations should be acknowledged. First, the cross-sectional design precludes determination of temporal relationships and causal inference; therefore, the observed indirect associations should not be interpreted as evidence of causal mediation. Second, participants were recruited consecutively from a single tertiary centre, which may limit the generalisability of the findings to other settings and populations. In addition, the inclusion of only participants with MMSE scores ≥ 24 was intended to support reliable completion of the patient-reported assessments; however, this criterion excluded stroke survivors with cognitive impairment and may further limit the generalisability of the findings to the broader post-stroke population, particularly individuals with mild or more pronounced cognitive impairment. Several potentially relevant confounding factors were not comprehensively assessed, including depressive symptoms, nutritional status, frailty, functional dependency, comorbidity burden, and stroke lesion location. Although oral pain or discomfort was recorded as part of the clinical data collection, it was not assessed using a detailed validated pain measure or included as an independent covariate in the present analyses. These unmeasured or incompletely characterized factors may have influenced both eating-related experiences and oral health-related quality of life and may therefore partly account for the observed associations. Residual confounding cannot be excluded. In addition, no formal correction for multiple comparisons was applied; therefore, the number of statistical tests performed may have increased the risk of type I error, particularly for secondary and exploratory analyses. The final sample size was slightly below the a priori target (100 vs. 109 participants); therefore, non-significant findings should be interpreted cautiously, as the study may have had limited power to detect smaller associations. This limitation may be particularly relevant to the exploratory indirect-effect analysis, for which the available sample may have provided insufficient power to detect smaller specific indirect effects. Accordingly, the absence of statistically supported specific indirect effects should not be interpreted as evidence that such associations are absent. Third, oral health-related quality of life was assessed using patient-reported outcome measures rather than objective clinical dental examinations. Detailed information on dental status, number of teeth, occlusal function, periodontal status, xerostomia and prosthesis quality was not available. Therefore, it cannot be determined to what extent the observed associations reflect underlying oral conditions, swallowing-related difficulties, psychosocial perceptions, or broader health status. Accordingly, the findings should not be interpreted as reflecting objective oral health status or the clinical severity of dental conditions. Finally, the inclusion criteria resulted in a relatively selected sample comprising participants with FOIS scores ≥ 4, MMSE scores ≥ 24 and sufficient communication abilities to complete the questionnaires independently. Consequently, individuals with more severe dysphagia, aphasia, cognitive impairment or exclusive tube feeding were not represented. This selection limits the external validity of the findings, which should primarily be considered applicable to cognitively and communicatively able stroke survivors with at least partial oral intake and should not be extrapolated to the broader stroke population, particularly those with more severe swallowing, cognitive or communication impairments.

Future multicentre longitudinal and interventional studies including more clinically diverse stroke populations and objective dental assessments are warranted to confirm these associations, clarify their temporal relationships and determine the potential clinical relevance of integrating swallowing-related, eating-related and oral health-related quality-of-life considerations in post-stroke care.

## 5. Conclusions

This study identified a significant total indirect association between functional oral intake and oral health-related quality of life through Desire to Eat, Food Selection, and Eating Time considered collectively in older adults with stroke. Functional oral intake and eating-related experiences were significantly associated with oral health-related quality of life, emphasizing the relevance of patient-reported eating experiences in this population. However, the cross-sectional design precludes determination of temporal or causal relationships, and the findings should therefore be interpreted as statistical associations rather than evidence of a causal mechanism. From a patient-centred perspective, eating-related experiences may warrant consideration alongside swallowing function and functional oral intake when evaluating oral health-related quality of life after stroke. Future prospective and interventional studies incorporating objective dental assessments are warranted to confirm these associations, clarify their temporal relationships, and determine their clinical relevance to post-stroke care.

## Figures and Tables

**Table 1:** Demographic, Clinical and Descriptive Characteristics of the Participants.

Variable	*n* (%)	Mean ± SD	Median (IQR)	Min–Max
**Sex**				
Female	49 (49.0)	–	–	–
Male	51 (51.0)	–	–	–
**Stroke type**				
Ischemic	59 (59.0)	–	–	–
Hemorrhagic	41 (41.0)	–	–	–
Age (years)	–	72.08 ± 7.36	72.00 (10.00)	65–90
Time since stroke (months)	–	5.20 ± 3.77	4.00 (5.00)	1–17
NIHSS	–	17.00 ± 9.36	16.00 (14.00)	1–40
FOIS	–	5.94 ± 1.20	6.00 (2.00)	4–7
**SWAL-QOL domains**				
Desire to Eat	–	63.66 ± 30.20	75.00 (50.00)	0–100
Food Selection	–	68.63 ± 25.47	75.00 (37.50)	0–100
Eating Time	–	65.63 ± 30.17	75.00 (50.00)	0–100
Fear	–	50.19 ± 33.13	50.00 (50.00)	0–100
Social	–	63.00 ± 27.99	62.50 (37.50)	0–100
GOHAI	–	36.62 ± 17.47	37.00 (29.00)	2–60
OHIP-14	–	25.30 ± 17.04	23.00 (27.00)	0–56

Data are presented as *n* (%) for categorical variables and as mean ± standard deviation (SD), median (interquartile range [IQR]), and minimum–maximum values for continuous and ordinal variables, as appropriate. Total sample size: *n* = 100. Abbreviations: FOIS, Functional Oral Intake Scale; GOHAI, Geriatric Oral Health Assessment Index; NIHSS, National Institutes of Health Stroke Scale; OHIP-14, Oral Health Impact Profile-14; SWAL-QOL, Swallowing Quality of Life Questionnaire. Higher SWAL-QOL and GOHAI scores indicate better quality of life, whereas higher OHIP-14 scores indicate poorer oral health-related quality of life.

**Table 2:** Pearson Correlations Between Oral Health-Related Quality-of-Life Measures and Eating-Related Quality of Life.

Measure	Variable	GOHAI			OHIP-14		
		r	95% CI	*p*	r	95% CI	*p*
**SWAL-QOL domains**	Desire to Eat	0.632	0.498 to 0.737	<0.001	−0.603	−0.713 to −0.464	<0.001
	Food Selection	0.468	0.301 to 0.606	<0.001	−0.503	−0.634 to −0.342	<0.001
	Eating Time	0.582	0.437 to 0.698	<0.001	−0.578	−0.695 to −0.432	<0.001
	Fear	0.582	0.437 to 0.698	<0.001	−0.502	−0.633 to −0.341	<0.001
	Social	0.419	0.240 to 0.570	<0.001	−0.426	−0.576 to −0.249	<0.001
**Oral health-related quality-of-life measures**	OHIP-14	−0.804	−0.865 to −0.720	<0.001	—	—	—

Pearson correlation coefficients (r), 95% confidence intervals (CIs), and corresponding *p* values are presented (*n* = 100). A *p* value < 0.05 was considered statistically significant. Higher GOHAI scores indicate better oral health-related quality of life, and higher SWAL-QOL domain scores indicate better swallowing-related quality of life, whereas higher OHIP-14 scores indicate poorer oral health-related quality of life. Abbreviations: CI, confidence interval; GOHAI, Geriatric Oral Health Assessment Index; OHIP-14, Oral Health Impact Profile-14; SWAL-QOL, Swallowing Quality of Life Questionnaire.

**Table 3:** Spearman Correlations Between Functional Oral Intake and Oral Health-Related Quality of Life/Eating-Related Quality-of-Life Domains.

Measure	Variable	FOIS		
		ρ	95% CI	*p*
**Oral health-related quality-of-life measures**	GOHAI	0.487	0.322 to 0.624	<0.001
	OHIP-14	−0.571	−0.690 to −0.423	<0.001
**SWAL-QOL domains**	Desire to Eat	0.504	0.344 to 0.635	<0.001
	Food Selection	0.306	0.116 to 0.474	0.002
	Eating Time	0.300	0.109 to 0.469	0.002
	Fear	0.365	0.181 to 0.524	<0.001
	Social	0.344	0.157 to 0.507	<0.001

Spearman’s rank correlation coefficients (ρ), 95% confidence intervals (CIs), and corresponding *p* values are presented (*n* = 100). A *p* value < 0.05 was considered statistically significant. Higher FOIS scores indicate better functional oral intake, higher GOHAI scores indicate better oral health-related quality of life, and higher SWAL-QOL domain scores indicate better swallowing-related quality of life, whereas higher OHIP-14 scores indicate poorer oral health-related quality of life. Abbreviations: CI, confidence interval; FOIS, Functional Oral Intake Scale; GOHAI, Geriatric Oral Health Assessment Index; OHIP-14, Oral Health Impact Profile-14; SWAL-QOL, Swallowing Quality of Life Questionnaire.

**Table 4:** Multiple Linear Regression Models of Factors Associated with Oral Health-Related Quality of Life.

Outcome	Variable	B	SE	β	t	*p*	95% CI for B	VIF
**GOHAI**	Constant	−11.013	15.751	—	−0.699	0.486	−42.300 to 20.274	—
	Age	0.078	0.165	0.034	0.470	0.639	−0.251 to 0.406	1.102
	NIHSS	−0.079	0.162	−0.042	−0.488	0.626	−0.400 to 0.242	1.546
	FOIS	2.397	1.266	0.164	1.894	0.061	−0.117 to 4.912	1.547
	Desire to Eat	0.144	0.064	0.249	2.244	0.027	0.017 to 0.272	2.537
	Food Selection	0.123	0.058	0.179	2.122	0.037	0.008 to 0.238	1.471
	Eating Time	0.165	0.054	0.285	3.069	0.003	0.058 to 0.272	1.776
	Fear	0.080	0.053	0.152	1.521	0.132	−0.025 to 0.185	2.062
	Social	−0.053	0.059	−0.084	−0.892	0.374	−0.170 to 0.065	1.841
**OHIP-14**	Constant	59.840	14.285	—	4.189	<0.001	31.465 to 88.216	—
	Age	0.136	0.150	0.062	0.906	0.367	−0.162 to 0.434	1.102
	NIHSS	0.277	0.147	0.152	1.892	0.062	−0.014 to 0.569	1.546
	FOIS	−4.324	1.148	−0.304	−3.766	<0.001	−6.604 to −2.043	1.547
	Desire to Eat	−0.086	0.058	−0.153	−1.483	0.142	−0.202 to 0.029	2.537
	Food Selection	−0.145	0.053	−0.217	−2.756	0.007	−0.249 to −0.040	1.471
	Eating Time	−0.169	0.049	−0.300	−3.470	0.001	−0.266 to −0.072	1.776
	Fear	−0.011	0.048	−0.022	−0.235	0.815	−0.106 to 0.084	2.062
	Social	0.060	0.054	0.098	1.117	0.267	−0.047 to 0.166	1.841

Unstandardized regression coefficients (B), standard errors (SEs), standardized regression coefficients (β), t statistics, corresponding *p* values, 95% confidence intervals (CIs) for the unstandardized regression coefficients (B), and variance inflation factor (VIF) values are presented (*n* = 100). Both multiple linear regression models were fitted using the Enter method. The GOHAI model was statistically significant (R^2^ = 0.558, adjusted R^2^ = 0.519, F(8, 91) = 14.377, *p* < 0.001; Durbin–Watson = 1.770). The OHIP-14 model was also statistically significant (R^2^ = 0.618, adjusted R^2^ = 0.584, F(8, 91) = 18.403, *p* < 0.001; Durbin–Watson = 1.408). Statistical significance was set at *p* < 0.05. Higher GOHAI scores indicate better oral health-related quality of life, whereas higher OHIP-14 scores indicate poorer oral health-related quality of life. Higher FOIS and SWAL-QOL domain scores indicate better functional oral intake and swallowing-related quality of life, respectively. Abbreviations: CI, confidence interval; FOIS, Functional Oral Intake Scale; GOHAI, Geriatric Oral Health Assessment Index; NIHSS, National Institutes of Health Stroke Scale; OHIP-14, Oral Health Impact Profile-14; SE, standard error; SWAL-QOL, Swallowing Quality of Life Questionnaire; VIF, variance inflation factor.

**Table 5:** Exploratory Indirect-Effect Analysis of the Association Between FOIS and GOHAI Through Eating-Related Quality-of-Life Domains (PROCESS Model 4).

Path/Effect	B	SE/BootSE	t	95% CI	*p*
**FOIS → Desire to Eat (a_1_)**	7.6166	2.3618	3.225	2.928 to 12.306	0.002
**FOIS → Food Selection (a_2_)**	6.3432	2.0560	3.085	2.262 to 10.424	0.003
**FOIS → Eating Time (a_3_)**	6.2194	2.3912	2.601	1.474 to 10.965	0.011
**Desire to Eat → GOHAI (b_1_)**	0.1824	0.0598	3.050	0.064 to 0.301	0.003
**Food Selection → GOHAI (b_2_)**	0.1317	0.0655	2.011	0.002 to 0.262	0.047
**Eating Time → GOHAI (b_3_)**	0.0862	0.0609	1.415	−0.035 to 0.207	0.160
**Total effect (c)**	4.8679	1.4390	3.383	2.012 to 7.723	0.001
**Direct effect (c′)**	2.1183	1.3126	1.614	−0.486 to 4.722	0.110
**Total indirect effect**	2.7496	1.0752 *	—	0.6070 to 4.9102	—
Desire to Eat specific indirect effect	1.3894	0.7689 *	—	−0.0187 to 2.9981	—
Food Selection specific indirect effect	0.8354	0.5517 *	—	−0.0564 to 2.0769	—
Eating Time specific indirect effect	0.5248	0.4356 *	—	−0.2075 to 1.5016	—

Unstandardized regression coefficients (B), standard errors (SEs), t statistics, 95% confidence intervals (CIs), and corresponding *p* values are presented (*n* = 100). The exploratory indirect-effect analysis was performed using PROCESS Model 4, with Desire to Eat, Food Selection, and Eating Time entered simultaneously as parallel intermediate variables. Indirect effects were estimated using 5000 bias-corrected bootstrap samples and are presented with bootstrap standard errors (BootSE) and 95% bootstrap confidence intervals. An indirect effect was considered statistically supported when the 95% bootstrap confidence interval did not include zero. Given the cross-sectional design, the results represent statistical indirect associations and should not be interpreted as evidence of causal mediation or temporal pathways. Abbreviations: BootSE, bootstrap standard error; CI, confidence interval; FOIS, Functional Oral Intake Scale; GOHAI, Geriatric Oral Health Assessment Index; SE, standard error. * *p* ≤ 0.05.

## Data Availability

The data presented in this study are available on request from the corresponding author due to privacy and ethical restrictions.

## Supplementary Materials

The following supporting information can be downloaded at: https://www.mdpi.com/article/10.3390/healthcare14182970/s1, Table S1. STROBE Statement—checklist of items that should be included in reports of observational studies.
