# Supplementary material for: Functional Oral Intake, Eating-Related Quality of Life and Oral Health-Related Quality of Life in Older Adults with Stroke: A Cross-Sectional Study
Source: Healthcare (Basel). 2026 Sep 11;14(18):2970. doi: 10.3390/healthcare14182970 (PMC13606482; doi:10.3390/healthcare14182970)
Supplement: Supplementary file 1 [file healthcare-14-02970-s001.zip › healthcare-4525302-supplementary.pdf]

Functional Oral Intake, Eating-Related Quality of Life and Oral Health-Related Quality of Life in Older Adults with Stroke: A Cross-Sectional Study

Table S1. STROBE Statement—checklist of items that should be included in reports of observational studies

	No.	Recommendation	Reported in manuscript / status
**Title and abstract**	1	(*a*) Indicate the study's design with a commonly used term in the title or the abstract	Title (p. 1): identifies the report as "A Cross-Sectional Study".
		(*b*) Provide in the abstract an informative and balanced summary of what was done and what was found	Abstract (pp. 1–2): provides an informative, balanced summary of the background/objective, cross-sectional methods, n = 100 sample, measures, correlation/regression/exploratory indirect-effect analyses, principal findings with effect estimates and 95% CIs, and cautious conclusions.
**Introduction**			
Background/rationale	2	Explain the scientific background and rationale for the investigation being reported	Introduction (pp. 2–3): describes the clinical and scientific rationale linking functional oral intake, eating-related experiences, and oral health-related quality of life after stroke, and identifies the evidence gap.
Objectives	3	State specific objectives, including any prespecified hypotheses	Introduction, final paragraphs (p. 3): states the primary and secondary objectives, prespecified hypotheses, and the exploratory indirect-effect analysis; explicitly notes that the cross-sectional analysis cannot establish temporal or causal mediation.
**Methods**			
Study design	4	Present key elements of study design early in the paper	Methods, 2.1 Study Design and Setting (p. 3): presents the single-centre cross-sectional observational design, outpatient setting, consecutive recruitment, and single-visit assessment early in the Methods.
Setting	5	Describe the setting, locations, and relevant dates, including periods of recruitment, exposure, follow-up, and data collection	Methods, 2.1 Study Design and Setting (p. 3) and 2.2 Study Population (pp. 3–4): Istanbul Atlas University Hospital Neurology and Physical Medicine and Rehabilitation outpatient clinics; consecutive recruitment and data collection from April to July 2026 during one study visit. Follow-up: Not applicable.
Participants	6	(*a*) *Cohort study*—Give the eligibility criteria, and the sources and methods of selection of participants. Describe methods of follow-up<br>*Case-control study*—Give the eligibility criteria, and the sources and methods of case ascertainment and control selection. Give the rationale for the choice of cases and controls<br>*Cross-sectional study*—Give the eligibility criteria, and the sources and methods of selection of participants	Methods, 2.1–2.3 (pp. 3–4): reports consecutive recruitment, clinical/radiological confirmation of first-ever ischemic or haemorrhagic stroke, source clinics, age ≥65 years, EAT-10 ≥3, FOIS ≥4, MMSE ≥24, written consent, and all exclusion criteria.
		(*b*) *Cohort study*—For matched studies, give matching criteria and number of exposed and unexposed<br>*Case-control study*—For matched studies, give matching criteria and the number of controls per case	Not applicable: this cross-sectional study did not use matching.

	No.	Recommendation	Reported in manuscript / status
Variables	7	Clearly define all outcomes, exposures, predictors, potential confounders, and effect modifiers. Give diagnostic criteria, if applicable	Methods, 2.2–2.5 (pp. 3–6): defines the study population and eligibility criteria; demographic/clinical variables; stroke severity (NIHSS); functional oral intake (FOIS); selected SWAL-QOL domains; oral health-related quality of life outcomes (GOHAI and OHIP-14); predictors and covariates used in the regression and exploratory models.
Data sources/ measurement	8*	For each variable of interest, give sources of data and details of methods of assessment (measurement). Describe comparability of assessment methods if there is more than one group	Methods, 2.3 Data Sources and 2.4 Assessment Procedures (pp. 4–5): describes the standardized participant form, face-to-face paper administration, participant self-report without proxy assistance, assessors, scoring, ranges, interpretation, and Turkish validity/reliability of FOIS, SWAL-QOL, GOHAI, and OHIP-14.
Bias	9	Describe any efforts to address potential sources of bias	Methods, 2.1–2.4 (pp. 3–5): consecutive recruitment, predefined eligibility criteria, standardized single-visit procedures, two experienced assessors, direct participant responses, and immediate completeness review were used to reduce selection, measurement, and missing-data bias. Discussion (pp. 10–12) addresses residual confounding, construct overlap, single-centre selection, and measurement limitations.
Study size	10	Explain how the study size was arrived at	Methods, 2.5 Statistical Analysis (p. 5): a priori G*Power calculation for multiple linear regression with eight predictors, f^2 = 0.15, two-sided α = 0.05, and 80% power produced a target of n = 109; the final sample was n = 100 because of eligible-participant availability during the predefined recruitment period.
Quantitative variables	11	Explain how quantitative variables were handled in the analyses. If applicable, describe which groupings were chosen and why	Methods, 2.4–2.5 (pp. 4–6): continuous variables were summarized using mean ± SD and median (IQR)/range as appropriate; FOIS was treated as ordinal for Spearman correlations; GOHAI, OHIP-14, and SWAL-QOL domains were analysed as continuous scores. No quantitative variables were categorized for the main analyses.
Statistical methods	12	(*a*) Describe all statistical methods, including those used to control for confounding	Methods, 2.5 Statistical Analysis (pp. 5–6): describes descriptive statistics, distribution checks, Pearson/Spearman correlations with 95% CIs, separate Enter-method multiple linear regression models adjusting for age, NIHSS, FOIS, Desire to Eat, Food Selection, Eating Time, Fear, and Social, and exploratory PROCESS Model 4 analysis with 5,000 bias-corrected bootstrap samples. Regression coefficients, 95% CIs, assumptions, VIF/tolerance, model fit, and two-sided significance threshold are specified.
		(*b*) Describe any methods used to examine subgroups and interactions	Methods, 2.5 (p. 6) and Results, 3.5 (pp. 9–10): exploratory parallel indirect-effect analysis examined Desire to Eat, Food Selection, and Eating Time simultaneously as intermediate variables. Subgroup and interaction analyses: Not applicable/not performed.

	No.	Recommendation	Reported in manuscript / status
		(*c*) Explain how missing data were addressed	Methods, 2.3 (p. 4) and 2.5 (p. 5): forms were checked for completeness during the visit; there were no missing data in analysed variables, so no imputation was required.
		(*d*) *Cohort study*—If applicable, explain how loss to follow-up was addressed<br>*Case-control study*—If applicable, explain how matching of cases and controls was addressed<br>*Cross-sectional study*—If applicable, describe analytical methods taking account of sampling strategy	Methods, 2.1–2.2 and 2.5 (pp. 3–6): consecutive recruitment from the two specified outpatient clinics is described. No weighting, clustering, stratification, matching, or follow-up adjustment was required for this single-centre cross-sectional sample.
		(*e*) Describe any sensitivity analyses	Not applicable: no sensitivity analyses were performed. The a priori power calculation is reported under item 10 (Methods, p. 5).
**Results**			
Participants	13*	(a) Report numbers of individuals at each stage of study—eg numbers potentially eligible, examined for eligibility, confirmed eligible, included in the study, completing follow-up, and analysed	Methods, 2.2 Study Population (pp. 3–4) and Results, 3.1 (p. 6): 100 eligible, consecutively recruited participants were included and analysed. Numbers assessed before eligibility are not reported. Follow-up: Not applicable.
		(b) Give reasons for non-participation at each stage	Not reported: the manuscript does not provide numbers or reasons for non-participation before enrolment.
		(c) Consider use of a flow diagram	Not applicable/not included: there were no longitudinal follow-up stages; a participant flow diagram was not provided.
Descriptive data	14*	(a) Give characteristics of study participants (eg demographic, clinical, social) and information on exposures and potential confounders	Results, 3.1 Participant Characteristics and Table 1 (pp. 6–7): reports n = 100, age, sex, stroke type, time since stroke, NIHSS, FOIS, selected SWAL-QOL domains, GOHAI, and OHIP-14; Methods, 2.4.1 (p. 4) lists additional collected demographic and clinical characteristics.
		(b) Indicate number of participants with missing data for each variable of interest	Methods, 2.5 (p. 5): no analysed variable had missing data. Table 1 and all analysis tables report n = 100 (pp. 6–10).
		(c) *Cohort study*—Summarise follow-up time (eg, average and total amount)	Not applicable: this was not a cohort study and had no follow-up period.
Outcome data	15*	*Cohort study*—Report numbers of outcome events or summary measures over time	Not applicable: cohort outcome events or longitudinal outcomes were not assessed.
		Case-control study—Report numbers in each exposure category, or summary measures of exposure	Not applicable: the study was not a case-control study and did not classify participants into exposure categories.
		Cross-sectional study—Report numbers of outcome events or summary measures	Results, 3.1 and Table 1 (pp. 6–7): reports summary measures for all participant, clinical, functional oral intake, eating-related quality-of-life, and oral health-related quality-of-life variables in the cross-sectional sample (n = 100).
Main results	16	(*a*) Give unadjusted estimates and, if applicable, confounder-adjusted estimates and their precision (eg, 95% confidence interval). Make clear which confounders were adjusted for and why they were included	Results, 3.2–3.5 and Tables 2–5 (pp. 7–10): reports unadjusted Pearson/Spearman associations with 95% CIs; adjusted Enter-method regression coefficients (B and β), 95% CIs for B, VIFs, R^2, adjusted R^2, F tests, p values, and model diagnostics; and exploratory total/specific indirect effects with 95% bootstrap CIs. Covariates are identified in Methods, 2.5 (pp. 5–6).

	No.	Recommendation	Reported in manuscript / status
		(*b*) Report category boundaries when continuous variables were categorized	Not applicable: continuous variables were not categorized in the reported analyses.
		(*c*) If relevant, consider translating estimates of relative risk into absolute risk for a meaningful time period	Not applicable: relative risks and time-dependent absolute risks were not estimated.
Other analyses	17	Report other analyses done—eg analyses of subgroups and interactions, and sensitivity analyses	Results, 3.5 and Table 5 (pp. 9–10): reports exploratory total and specific indirect effects through three parallel intermediate variables with 5,000 bias-corrected bootstrap samples and 95% CIs. Subgroup, interaction, and sensitivity analyses were not performed.
**Discussion**			
Key results	18	Summarise key results with reference to study objectives	Discussion, opening paragraph (p. 10), and Conclusion (pp. 12–13): summarize the main correlations, independent regression associations, and supported total indirect association in relation to the prespecified and exploratory objectives.
Limitations	19	Discuss limitations of the study, taking into account sources of potential bias or imprecision. Discuss both direction and magnitude of any potential bias	Discussion (pp. 10–12), especially limitations (p. 12): addresses cross-sectional temporality/causality, single-centre consecutive sampling, MMSE/aphasia/FOIS restrictions, unrecorded lesion location, unmeasured depression, lack of a dedicated validated oral/orofacial pain measure, multiple-testing type I error, final n below target and reduced power for smaller effects, absence of objective dental examinations, construct overlap, and limits on interpretation and generalisability.
Interpretation	20	Give a cautious overall interpretation of results considering objectives, limitations, multiplicity of analyses, results from similar studies, and other relevant evidence	Discussion (pp. 10–12) and Conclusion (pp. 12–13): provides a cautious interpretation in light of objectives, prior evidence, bidirectionality, clinical relevance, model findings, exploratory analysis, multiplicity, and study limitations; explicitly avoids causal mediation claims.
Generalisability	21	Discuss the generalisability (external validity) of the study results	Discussion, limitations and future directions (p. 12): explains that single-centre recruitment and exclusions (MMSE <24, severe communication impairment, FOIS <4/exclusive tube feeding) limit generalisability, and recommends multicentre studies with more clinically diverse stroke populations.
**Other information**			
Funding	22	Give the source of funding and the role of the funders for the present study and, if applicable, for the original study on which the present article is based	Funding (p. 13): the research received no external funding; an external funder role is therefore Not applicable.

*Give information separately for cases and controls in case-control studies and, if applicable, for exposed and unexposed groups in cohort and cross-sectional studies.

Note: An Explanation and Elaboration article discusses each checklist item and gives methodological background and published examples of transparent reporting. The STROBE checklist is best used in conjunction with this article (freely available on the Web sites of PLoS Medicine at http://www.plosmedicine.org/, Annals of Internal Medicine at http://www.annals.org/, and Epidemiology at http://www.epidem.com/). Information on the STROBE Initiative is available at www.strobe-statement.org.
